# Mitochondrial transplantation as a novel therapeutic strategy for cardiovascular diseases

**DOI:** 10.1186/s12967-023-04203-6

**Published:** 2023-05-25

**Authors:** Mingchu Sun, Wenhua Jiang, Nan Mu, Zihui Zhang, Lu Yu, Heng Ma

**Affiliations:** 1 Institute of Medical Research, Northwestern Polytechnical University, Xi’an, 710072 Shaanxi P.R. China; 2 Department of Physiology and Pathophysiology, School of Basic Medical Sciences, Fourth Military Medical University, Xi’an, 710032 China; 3 Department of Pathology, Xijing Hospital, Fourth Military Medical University, Xi’an, 710032 China

**Keywords:** Cardiovascular diseases, Mitochondrial dysfunction, Mitochondrial transplantation, Ischemia reperfusion injury, Therapy

## Abstract

Cardiovascular disease (CVD) is the leading cause of noncommunicable disease-related death worldwide, and effective therapeutic strategies against CVD are urgently needed. Mitochondria dysfunction involves in the onset and development of CVD. Nowadays, mitochondrial transplantation, an alternative treatment aimed at increasing mitochondrial number and improving mitochondrial function, has been emerged with great therapeutic potential. Substantial evidence indicates that mitochondrial transplantation improves cardiac function and outcomes in patients with CVD. Therefore, mitochondrial transplantation has profound implications in the prevention and treatment of CVD. Here, we review the mitochondrial abnormalities that occur in CVD and summarize the therapeutic strategies of mitochondrial transplantation for CVD.

## Background

Cardiovascular diseases (CVD) are the leading cause of non-communicable disease-related deaths worldwide [1, 2]. With the gradually increased burden, CVD have become a major public health problem [2]. The diversity of risk factors, the complexity of pathological mechanisms, and the verity of comorbidities make the treatment of CVD even more challenging. Effective therapeutic strategies against CVD are urgently addressed.

Mitochondria play indispensable rolese in cardiovascular system. Mitochondrion is an energy center of the cardiomyocyte by constantly providing ATP. As one of the most complex and critical organelles in eukaryotic cells, mitochondria play an important role in cell signal transduction, redox balance, biotransformation of amino acids and lipids, calcium homeostasis, apoptosis and programmed cell death [3, 4] Intracellular energy balance is important for cardiomyocytes survival. Cardiomyocytes are one of the cell types with the highest content of mitochondria, and are highly dependent on mitochondrial oxidative phosphorylation to produce ATP. Mitochondria adapt quickly to changing environments to maintain metabolic homeostasis [5, 6]. Mitochondria dysfunction involves in the onset and development of CVD.

Nowadays, mitochondrial transplantation, an alternative treatment aimed at increasing mitochondrial number and improving mitochondrial function, has been emerged with great therapeutic potential [7]. Since protein components in mitochondria act as network hubs in multiple biological pathways are often affected simultaneously under pathological conditions, mitochondrial transplantation offers unique advantages over traditional pharmacological treatments targeting a single molecule [4]. Intracellular mitochondrial movement promotes the connection and formation of a dynamic mitochondrial network. In addition, the intercellular transfer of mitochondria was observed both in vitro and in vivo under physiological and pathophysiological conditions [3]. Since the dynamics and transferable capacity of mitochondria have been uncovered gradually, the therapeutic potential of mitochondrial transplantation for CVD as a rapidly developing field has attracted great attention. Substantial evidence indicates that mitochondrial transplantation improves cardiac function and outcomes in patients with CVD, and mitochondrial transplantation has profound implications in the prevention and treatment of CVD. Here we review the mitochondrial abnormalities that occur in CVD and summarize the therapeutic strategies of mitochondrial transplantation for CVD.

## The role of mitochondria in cardiovascular system

Mitochondria, which are widely regarded as the “energy hub” of cells, play major roles in maintain metabolism and intracellular Ca^2+^ homeostasis, regulation of inflammatory reactions and molecular signaling.

### Key signal pathways in mitochondria

AMP-activated protein kinase (AMPK) pathway is one of the mitochondria specific signaling pathways, which is activated when the adenosine monophosphate (AMP)/ATP ratio increases. AMPK has been implicated in the energy balance of key enzymes and regulatory nodes in various metabolic pathways, such as lipid and glucose metabolism, mitochondrial dynamics, autophagy, and protein synthesis [8]. In addition, the ratio of nicotinamide adenine dinucleotide (NAD) to NADH can be used as another indicator of mitochondrial metabolism, perceiving and transmitting the metabolic state of mitochondria. Act as cofactors for many metabolic reactions, NAD levels vary with metabolic activity. At the same time, it is also a family of proteases such as CD38, sirtuins protein (1–7) deacetylase and deacylase [9]. Reactive oxygen species (ROS) is a toxic by-product of mitochondrial dysfunction. The decrease of NAD level stably mediated by hypoxia inducible factor-1α is harmful to mitochondrial function in aged mice [10].

**Fig. 1:**
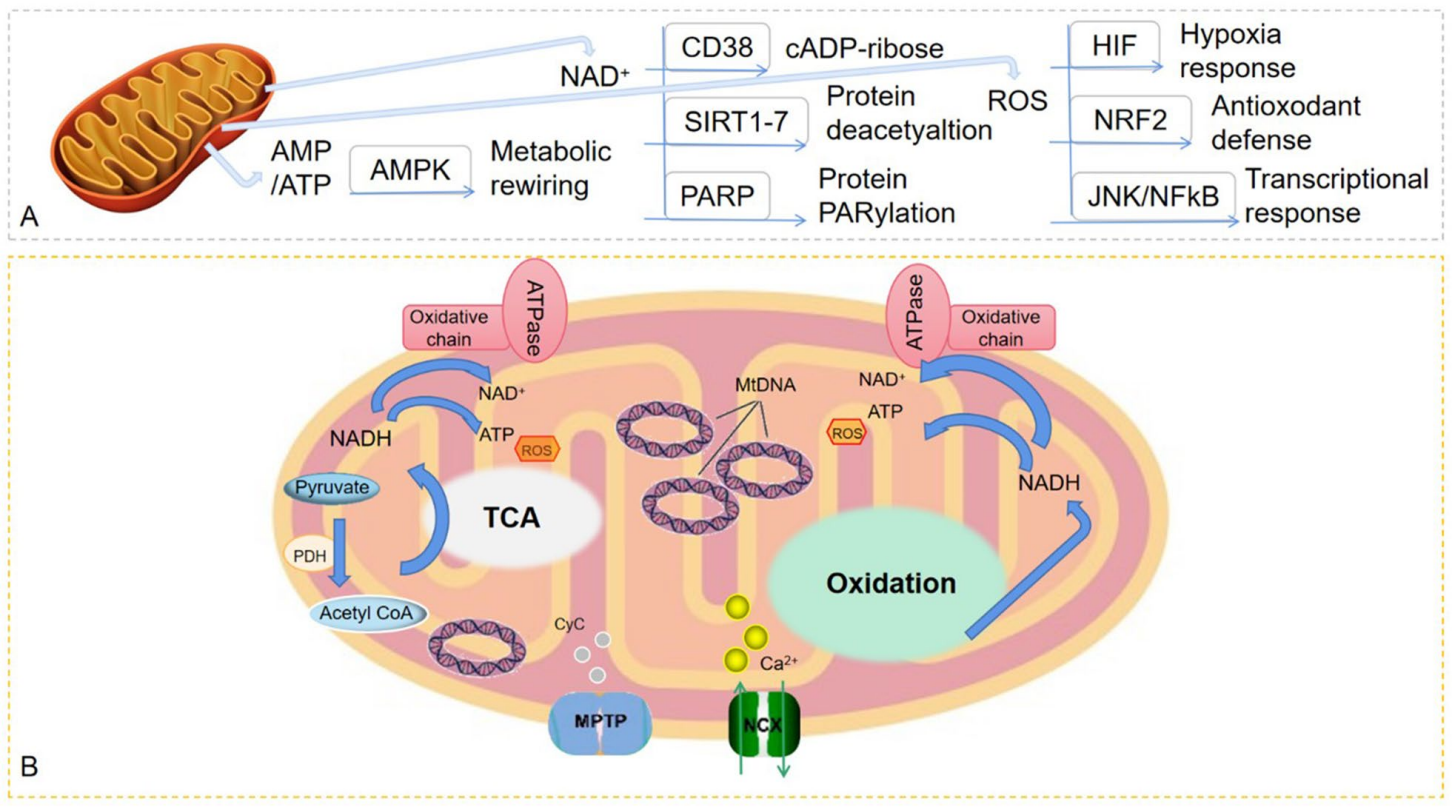
Key signal pathways of mitochondrial metabolism. **A** Major mitochondrial metabolites and cellular signaling pathways. **B** Main factors and pathways involved in mitochondrial metabolism under physiological conditions of cardiovascular system

The main process of mitochondrial metabolism is that sugars, lipids, and proteins are oxidized through tricarboxylic acid cycle and oxidative phosphorylation, which involves a series of enzymatic reactions to provide energy for cardiomyocytes [11]. Under physiological conditions, compared with glycolysis, oxidative phosphorylation is more effective way to produce adenosine triphosphate (ATP). Intracellular calcium homeostasis is disrupted under stress, reducing oxidative phosphorylation efficiency and ATP production, producing excessive ROS, which further causes mitochondrial dysfunction and ultimately leads to the development of CVD (Fig. 1).

### Mitochondria dysfunction and CVD

Mitochondrial dysfunction leads to increased ROS, energy stress, and cell death, which are closely related to the onset and development of CVD [12].

Mitochondria abnormalities range from structure to function has been suggested in CVD [13]. Abnormalities in mitochondrial size and distribution, the most common manifestations of structural abnormalities, have been found in various CVD such as myocardial infraction, ischemia/reperfusion (I/R) injury, pulmonary hypertension, diabetes [14]. The two processes of fusion and fission occur simultaneously and continuously, and the number, shape, and distribution of mitochondria all influence the respiratory function of mitochondria [15, 16]. Ultrastructural analysis shows a significant increase in the number and size of mitochondria, resulting in the enlargement of cardiomyocytes and sarcomere disorders hinder contractile activity in mitochondrial heart disease [17].

The mitochondrial respiratory chain is also a major source of intracellular ROS while providing energy. Conversely, ROS can also affect the activities of respiratory chain complexes III and IV [15]. Mitochondrial ROS burst contributes to cardiomyocyte death, endothelial dysfunction and vascular occlusion in myocardial I/R injury [18, 19]. ROS causes mitochondrial Ca^2+^ overload, which in turn further increases ROS levels, this feedback loop maintains a Ca^2+^overload state with increased ROS production [20].

The nonspecific high conductance channels of mitochondrial permeability transition pore (mPTP) mediate the transition of mitochondrial permeability. Opening of mPTP leads to collapse of mitochondrial membrane potential, resulting in uncoupling of oxidative phosphorylation and ATP consumption [6, 21]. Related studies have shown that changes in mitochondrial permeability are associated with heart failure, hemorrhagic shock, hypertension, cardiomyopathy and cardiac I/R injury [14, 15, 22, 23]. In ischemic heart, Oxygen deprivation decrease oxidative phosphorylation in mitochondria, aerobic glycolysis becomes the primary mode of energy supply. Mitochondrial Ca^2+^ overload occurs along with increased ROS production and mPTP opening in ischemic cardiomyocytes [24].

Mitophagy is a defense behavior to restore mitochondrial components and selectively removes the accumulation of abnormal mitochondria, ensure energy supply and maintain cellular homeostasis [25]. Mitophagy abnormalities are closely related to a variety of diseases, especially CVD, including sepsis-induced myocardial dysfunction [21], myocardial I/R injury [15], cardiomyopathy [25], heart failure, atherosclerosis, myocardial infarction and hypertrophy and diabetic cardiomyopathy [26].

### Mitochondrial transfer between cells and spontaneous mitochondrial transfer

Intercellular mitochondrial transfer is a form of intercellular interaction [27]. Mitochondrion derived proteins were initially discovered in exocrine proteomics, and subsequent studies further suggest that the secretion of damaged mitochondria by vesicles may be a mitochondrial quality control mechanism. Importantly, the transfer of healthy mitochondria from mesenchymal stem cells to target organs.

In 2006, Spees et al. demonstrated that wild mitochondria from bone marrow stem cells can be transferred to parenchymal cells displaying mitochondrial dysfunction to increase the aerobic respiration capacity of recipient mitochondria [28]. Although some studies have shown that exogenous mitochondria released from donor cells due to the delivery of autophagy may be degraded or integrated by recipient cells, the biological role of this interaction remains controversial [29, 30]. Accumulating evidence suggests that intercellular mitochondrial transfer occurs spontaneously in the cardiovascular system to maintain tissue homeostasis and development [7, 31]. In addition to bidirectional mitochondrial transfer between cardiomyocytes and fibroblasts [7], cardiomyocytes, vascular smooth muscle cells, and endothelial cells can act as donors or receptors during mitochondrial transfer [4].

Intercellular mitochondrial transfer has been demonstrated, but how exogenous mitochondria are internalized and integrated into the mitochondrial network of recipient cells is a key question in mitochondrial transplantation studies. Masuzawa et al. detected that mitochondria did not co-locate with any lysosomal or autophagosomal, indicating the internalization of exogenous mitochondria [32]. Later studies further revealed that mitochondrial internalization was actin-dependent in cardiomyocytes [33]. Many lines of evidence have demonstrated that the dynamic features of mitochondria are not restricted by cell boundaries, and the transfer of mitochondria between cells can integrate into the endogenous mitochondrial network of recipient cells to restore their biological functions [29, 34].

## Strategy and mechanism of mitochondrial transfer in CVD

Methods for the treatment of CVD by mitochondrial transplantation include naked mitochondrial transplantation, cell-mediated approaches including tunnel nanotubes (TNTs), cell fusion, and extracellular vehicles (EVs) [3]. There are also gap junction channels-mediated synaptic complexes and dendritic networks that are used in other diseases but have not been reported in CVD [35, 36]. Since then, a number of studies have shown mitochondrial engraftment by TNTs and cell fusion, which are summarized in Table 1.

**Table 1:** Summary of mitochondrial transfer between MSCs and recipient cells of heart

Donors	Condition	Recipients	Mechanism	Outcome	References
Human endothelial progenitor cells	Ex vivo	Rat cardiomyocytes	TNTs	The formation of intercellular junctions	Koyanagi et al. [46]
Human MSCs	In vivo	Rat cardiomyocytes	TNTs	Cell-to-cell crosstalk between MSCs and cardiomyocytes in co-culture	Plotnikov et al. [47]
MSCs	In vitro	Rat cardio-myoblasts	TNTs and cell fusion	Preserved cardio-myoblasts bioenergetics	Cselenyák et al. [48]
hBM-MSCs	In vitro	Adult mouse cardiomyocytes	Cell fusion	Metabolic reprogramming, transformation to progenitor state	Acquistapace et al. [49]
hMSCs	In vitro	Human vascular smooth muscle cells	TNTs	increased MSC proliferation	Vallabhaneni et al. [50]
hBM-MSCs	In vitro	Human umbilical vein endothelial cells	TNTs	Increased mitochondria biogenesis, decreased cell apoptosis, increased proliferation and finally promoted cell survival	Liu et al. [42]
BM-MSCs and iPSC-MSCs	In vivo	Mouse cardiomyocytes (doxorubicin-induced damage)	TNTs	Increased ATP production and mitochondria biogenesis, increased cell viability and decreased apoptosis	Zhang et al. [51]
BM-MSCs	In vitro	Rat cardiomyocytes	TNTs	Restored mitochondrial function, decreased cell apoptosis	Han et al. [52]
hMADSs	In vitro and in vivo	Cardiomyocytes and endothelial cells	TNTs	Increased mitochondrial biogenesis, decreased cell apoptosis	Mahrouf-Yorgov et al. [53]

*BM-MSCs* bone marrow mesenchymal stromal cells, *hBM-MSCs* human bone marrow mesenchymal stromal cells, *iPSC-MSCs* pluripotent stem cell-derived mesenchymal stem cells, *hMADS* human multipotent adipose-derived stem cells

### Naked mitochondrial transplantation

Naked mitochondrial transplantation is a technique in which mitochondria isolated from healthy tissue or cells are injected directly into the myocardium or into coronary arteries or veins with the aid of microinjection techniques to improve cardiac function [37–39]. Interestingly, cell-free mitochondria were reported to be present in human blood, although they have no potential for oxidative phosphorylation and are unlikely to be functional in vivo [40]. This method has no risk of complications such as autoimmune reaction, microvascular occlusion, arrhythmia, and intramyocardial hematoma, but it is technically demanding and has low efficacy [4].

### TNTs

Cell based mitochondrial transplantation can inject mesenchymal stem cells and progenitor cells into the myocardium. The main advantage of this technique is that mesenchymal stem cells can be derived from multiple tissues with high quality mitochondria [7]. TNTs is a transient filamentous membrane connected cell comprising cell membranes, F-actin, myosin, and tubulin, and studies have shown it to be a novel type of intercellular communication between neonatal rat cardiomyocytes and endothelial progenitor cells [41]. Mesenchymal stem cells (MSCs) rescued injured endothelial cells in an in vitro I/R model via tunnel structure mediated mitochondrial transfer [42]. In I/R injury and anthracycline induced cardiomyopathy, transplantation of MSCs mitochondria to endothelial cells enhances aerobic respiration to protect cardiomyocytes from oxidative stress, limit left ventricular dilatation and myocardial fibrosis [42–44]. As early as in 2005 it was shown that metabolic reprogramming can improve mitochondrial transfer in cardiac tissue.

The extension of TNTs required cell division control protein 42 homolog (CDC42), a cytoskeletal regulatory protein that controls the protrusion and growth [45]. M-Sec promoted nanotubes formation by enhancing the expression of epidermal growth factor receptor and activating the mammalian target of rapamycin (mTOR)/CDC42 signal transduction pathway, thereby increasing the expression of p53 [54]. In addition, mitochondrial Rho GTPase 1 (Miro1), a tail anchored outer mitochondrial membrane protein, mediated the movement of mitochondria along TNTs [51, 55]. Miro1 bound to the docking protein TRAK1/2 to recruit the motor protein kinesin and initiate microtubule based mitochondrial movement [56]. Ahmad et al. showed that Miro1 regulated the movement of mitochondria from MSCs to recipient cells, and its overexpression enhanced mitochondrial transfer and therapeutic effects [57]. An in vitro model of simulated I/R model, bone marrow MSCs rescued injured cardiomyocytes by TNTs mediated mitochondrial transfer, which increased mitochondrial membrane potential, enhanced mitochondrial function, and decreased cardiomyocytes apoptosis [52]. Mitochondria isolated from human MSCs via nasal administration in mice achieved rapid cellular internalization and restored brain structure and function [58]. However, nasal delivery has not been applied to CVD.

### Cell fusion

Partial (temporary) or complete (permanent) cell fusion is the process of realizing the gradual sharing of organelle and cytoplasmic components by merging plasma membranes [59]. Acquistapace et al. studied the fusion of human bone marrow MSCs and human pluripotent adipose stem cells with partially mature mouse cardiomyocytes [49]. The co-culture of cardiomyocytes and human pluripotent adipose stem cells allowed the exchange of materials and mitochondria and facilitated cardiomyocytes reprogramming. Several studies have pointed out that MSCs rescued damaged cells through paracrine mechanisms, however, there are many safety concerns such as arrhythmia and microcirculation occlusion, which limited the application of this transplantation method [4].

### EVs

EVs, including exocrine bodies, microvesicles and apoptotic bodies, are cell secreted nanoscale bilayer vesicles that stably reside in the extracellular fluid and participate as important messengers for cell communication, migration and angiogenesis [60]. Exocrine itself is related to the occurrence and development of CVD. Recent studies suggested that inhibition of inflammatory response and exocrine led to inhibition of apoptosis and oxidative stress in sepsis-induced cardiomyopathy. In mammalian cells microvesicle biogenesis was found to be controlled by cluster of differentiation 38 and cyclic adenosine diphosphate ribose signaling, while entry of exogenous mitochondria was regulated by integrin induced Src/Syk signaling [61]. Most importantly, EVs-mediated mitochondrial transfer is part of a fundamental cell biological process that occurs in multiple tissues, which makes the use of EVs for mitochondrial transfer as a new hotspot. Ibáñez and Villena-Gutierrez [62] proposed a novel strategy of mitochondrial transplantation, whereby EVs secreted the mitochondria from human-induced pluripotent stem cell-derived cardiomyocytes to damaged cardiomyocytes, attenuating myocardial infarction and I/R injury. Intracoronary or intravenous administration of mitochondria improved cardiac contractility and prevented left ventricular remodeling in I/R injury [63]. Brestoff et al. isolated mitochondria from adipocytes and transferred them into cardiomyocytes via small EVs to ameliorate I/R injury [64]. The complex composition of different EVs makes them unique for various diseases, and this method has high mitochondrial stability with no risk of microvascular occlusion and arrhythmias [4].

In addition, EVs are natural cell-derived drug carriers. EVs with a particle size larger than 200 nm, that is medium-to-large extracellular vesicles (m/lEVs) are naturally transported mitochondria during biogenesis to improve the survival rate of injured recipient tissues, and effectively protect mitochondrial integrity and activity, prolonging their life span in the blood, thus making EVs a promising carrier [65]. A recent study found that activation of PGC-1 α make m/lEVs carry a higher mitochondrial load, but it is often expensive and time-consuming [66]. Studies aimed at expanding the production capacity of EV and m/lEVs and increasing the mitochondrial load will expand the clinical application of mitochondrial therapeutics. We further compared the safety, effectiveness and flexibility of mitochondrial transplantation through co-incubation, microinjection, EVs mitochondrial delivery (Table 2).

**Table 2:** Different mitochondria transplantation methods in safety, efficacy and flexibility

Methods	Safety	Efficacy	Fexibility
Co-incubation	Low accuracy High risk of mitochondria damage	Moderate transfer efficiency mtDNA retention up to 12 passages	Reduced manipulation of large numbers of transplant recipient cells; easy to realize
Microinjection	Potentially harmful for the target, high risk of mitochondria damage	mtDNA is retained from 6–10 weeks after treatment	Limited number of cells transplanted
EVs	Mitochondrial and cellular integrity preservation	mtDNA retention not known	Low manipulation, easy to realize

Targeted mitochondrial transplantation is a challenging and promising task. For this reason, researchers are committed to finding ways to improve the efficiency of transplantation. Magneti or pressure driven techniques improved transplantation efficiency [66], and the application of optical tweezers achieved automated mitochondrial transplantation [67]. In addition, McCully and his group were granted a patent for an automated isolation of live mitochondrial device, which may be useful for the treatment of mitochondrial transplantation in CVD. Recently, a new delivery system was developed with the aid of a biocompatible polymer, that is, delivery by artificially encapsulating isolated mitochondria with a triphenylphosphine complex of dextran or a transactivates of transcribed glucan complexes [68, 69]. Mitochondria are almost completely contained in this delivery system, with high transfer efficiency and strong rescue ability.

**Fig. 2:**
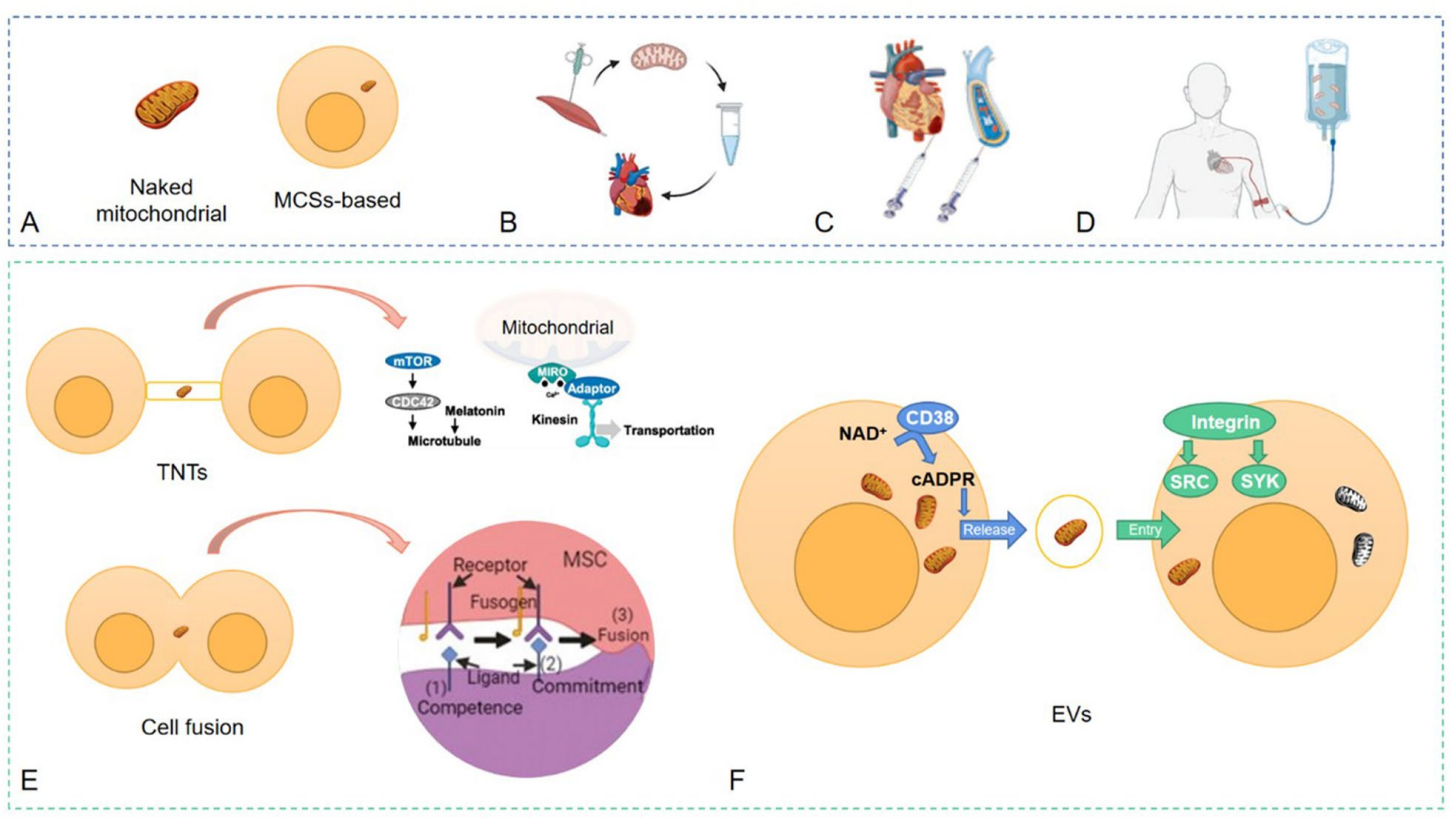
Strategy and mechanism of mitochondrial transfer in CVD. **A** Naked mitochondria or mitochondria carrying MSCs. **B** Mitochondrial transplantation overview. **C**, **D** Different delivery methods: mitochondria can either be transplanted through direct injection to the tissue of concern or through intravenous injection. **E** Cell-based mitochondrial transplantation. **F** Extracellular vehicles

Strategy and mechanism of mitochondrial transfer in CVD were summarized in Fig. 2. More feasible and possible strategic options will be available for mitochondrial transplantation in future studies.

## Therapeutic practices of mitochondrial transplantation for CVD

**Fig. 3:**
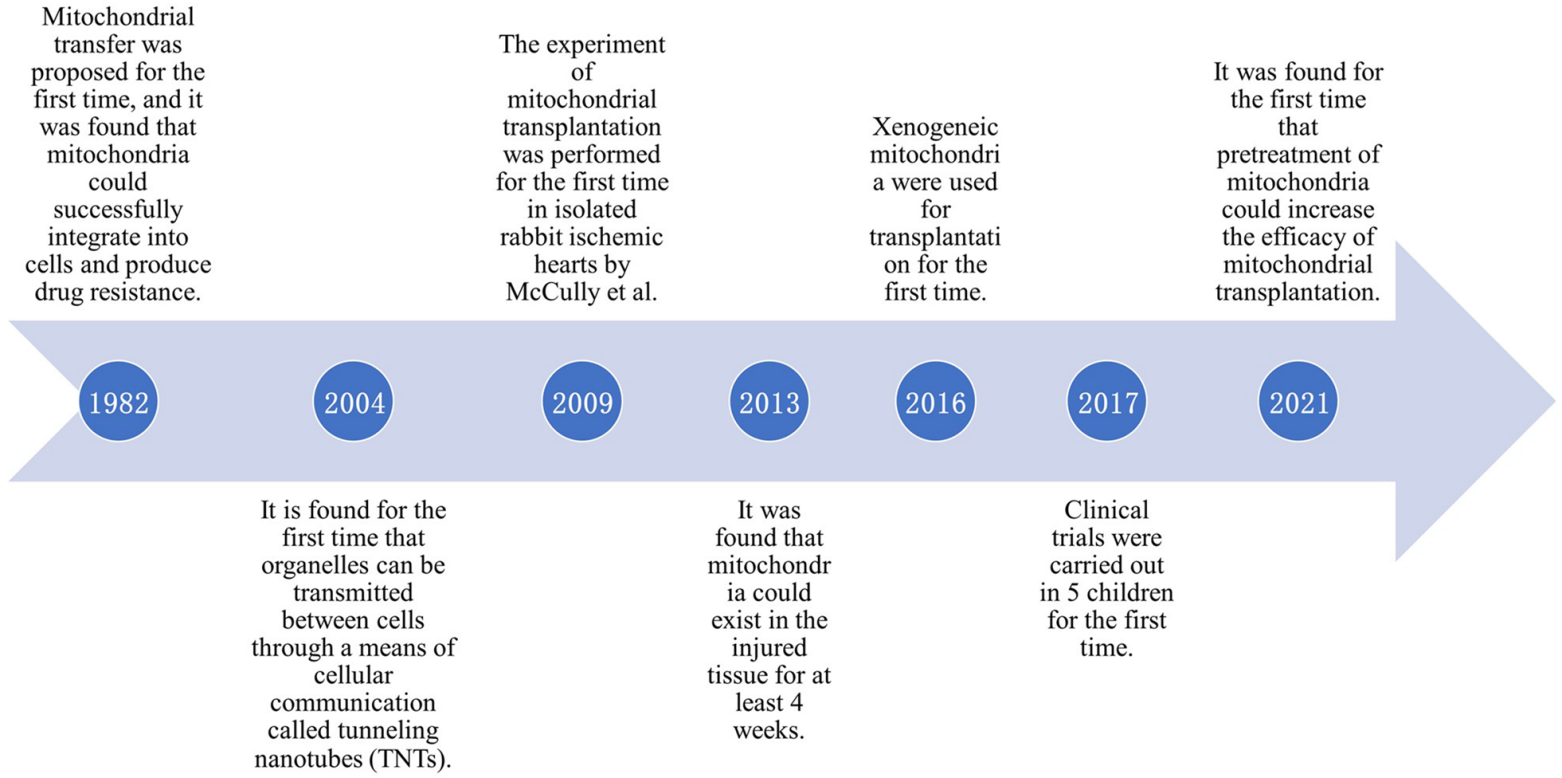
Important breakthroughs in the study of mitochondrial transplantation

Targeting important molecules involved in the mechanisms of mitochondrial dysfunction significantly improves cardiac function, implicating mitochondrial replacement therapy as an important approach for cardioprotection [70]. According to important breakthroughs in the study of mitochondrial transplantation (Fig. 3), more and more evidences prove that targeted mitochondrial transplantation is a promising strategy for the treatment of CVD.

### Myocardial I/R injury

The McCully group has been working on mitochondrial transplantation for the treatment of CVD. In their study, mitochondria isolated from the left ventricle of donor rabbit sham control or regional ischemia were injected directly into the ischemic site of the rabbit heart before reperfusion, it showed that mitochondria isolated from normal tissues significantly enhanced functional recovery and cell viability after ischemia [37]. Masuzawa et al. then successfully transplanted human mitochondria to a rabbit cardiac I/R model, achieving mitochondrial allotransplantation [32]. Their subsequent study transplanted exogenous mitochondria through the coronary vasculature and compared the outcomes of mitochondrial transplantation by two different routes of delivery. The concentration and distribution of mitochondria transported through the coronary arteries were higher than those achieved by direct mitochondrial injection in human fibroblasts [38]. Although both routes I/R injury, compared with safe, dense mitochondria injected in situ only near the administration site, vascular injection of mitochondria was less invasive and could be widely distributed over a short period of time. A study comparing the therapeutic effects of mitochondrial transplantation through coronary arteries before and during reperfusion, further demonstrated that coronary blood flow was temporarily restored and myocardial infarct size was reduced after mitochondrial transplantation [71]. Intracoronary implantation of mitochondria was further verified as a safe and effective method for the treatment of myocardial I/R injury, and ATP mediated vasodilation of inward potassium channels significantly increased coronary blood flow [72]. New implantation method improved the rate of mitochondrial uptake increasing the accuracy of mitochondrial distribution. However, serial multiple mitochondrial transplantation was not superior to single mitochondrial transplantation in a I/R injury model in Yorkshire swine, although both procedures significantly increased coronary blood flow and ejection fraction and reduced infarct size [73]. Mitochondria for transplantation can be derived from pectoralis major muscle cells as well as gastrocnemius myocytes. Isolation of allogeneic mitochondria from gastrocnemius muscles and injected them into coronary artery before harvesting donor heart showed prolonged cold ischemia time, enhanced graft function and reduced graft tissue injury [74]. These studies are summarized in Table 3.

**Table 3:** Research reports of mitochondrial transplantation for I/R injury

Species	Type of model	Mitochondrial sources	Injection sites and methods	Therapeutic outcomes	References
New Zealand white rabbits	Focal ischemia	Left ventricular	Direct injection into regional ischemia zone of the heart	Recovery of myocardial function	McCully et al. [37]
New Zealand white rabbits	Focal ischemia	Pectoralis major muscle tissues	Direct injection into regional ischemia zone of the heart	Reduced myocardial infarct size and enhanced regional myocardial function post-reperfusion	Masuzawa et al. [32]
New Zealand white rabbits	Global or regional ischemia	(1) xenograft: human cardiac fibroblasts (2) autograft: liver	Intracoronary injection (120 min after reperfusion)	Reduced infarct size and enhanced myocardial function	Cowan et al. [38]
Yorkshire pigs	Focal ischemia	pectoralis major muscle tissues	Direct injection under the endocardium (1 min before reperfusion)	No change in inflammatory and cytokine activation markers; decreased infarct size but no change in global function	Kaza et al. [71]
Yorkshire swine	Focal ischemia	Pectoralis major muscle tissues	Intracoronary injection (immediately on reperfusion)	Improved myocardial function, perfusion, and infarct size	Shin et al.[72]
Yorkshire pigs	Global ischemia	Pectoralis major muscle; swine cardiac fibroblast cell	Intracoronary injection: 15 min or 2 h post-reperfusion	Preserved myocardial function and oxygen consumption and, decreased infarct size	Guariento et al. [78]
Yorkshire pigs	Focal ischemia	Pectoralis major muscle tissues	Intracoronary injection (15 min before regional ischemia)	Reduced myocardial infarct size, improved myocardial function	Guariento et al. [73]
Yorkshire pigs	Focal ischemia	Pectoralis major muscle tissues	Direct injection into the left coronary ostium	Reduced myocardial infarct size and enhanced regional and global myocardial function post-reperfusion	Blitzer et al. [79]
C57BL/6	Focal ischemia	gastrocnemius muscle	Intracoronary injection (10 min before organ harvest and 5 min after transplantation)	Enhanced graft function and decreased graft tissue injury	Moskowitzova et al. [74]
Zucker Fatty rats	Global ischemia	Pectoralis major muscle tissues	Delivery to the coronary arteries via the aortic cannula	Recovery of left ventricular function, reduction of infarct size and area at risk	Doulamis et al. [76]
C57BL/6	Focal ischemia	Not reported	Direct injection at myocardium of the left ventricle	Inhibition of cardiomyocyte apoptosis in vitro	Sun et al. [39]

### Cardiomyopathy

Diabetes cardiomyopathy is the most common cardiovascular complication of diabetes [23]. The insulin secretion of islet β cells is mainly related to the production of mitochondrial ATP stimulated by glucose. It has been found that human adipose MSCs transferred mitochondria to human islet β cells under symbiotic conditions to enhance the bioenergy and insulin secretion capacity of damaged β cells [75], implying mitochondrial transplantation as promising for the treatment of diabetes. Doulamis IP et al. also demonstrated that mitochondrial transplantation mitochondrial transplantation improves diabetic myocardial function [76]. These studies suggest that mitochondrial transplantation is a promising therapeutic strategy to reduce cardiac damage in type 2 diabetes.

It also showed that mitochondrial transplantation was more effective in treating sepsis-induced cardiomyopathy than simply improving mitochondrial function [22]. Recently, Mokhtari B et al. have demonstrated that mitochondrial transplantation improved the mitochondrial function, biogenesis and kinetics related to SIRT-1/PGC-1 α network to prevent myocardial dysfunction induced by sepsis [77]. Anthracycline induced cardiomyopathy is a dose-dependent progressive myocardial injury, after intramyocardial injection mitochondria derived from human induced pluripotent stem cell derived MSCs to neonatal mouse cardiomyopathy model induced by anthracycline, both mitochondrial respiration and cardiomyocyte viability were significantly enhanced [51]. These results provide evidence that mitochondrial transplantation improved anthracycline-induced cardiomyopathy.

Mitochondrial cardiomyopathies are structural or functional abnormalities of the myocardium caused by defects in nuclear DNA or mtDNA genes and are usually characterized by hypertrophic cardiomyopathy, dilated cardiomyopathy, and cardiac conduction defects [80]. Potential treatments are to transplant or deliver entirely new mitochondria from healthy cells to diseased cells to improve outcomes in patients with acquired or congenital mitochondrial defects due to mitochondrial mutations [44]. Recently, Park et al. proposed that mitochondrial transplantation was a valuable strategy for the treatment of a variety of mitochondrial diseases [81], but further studies are needed to prove its therapeutic efficacy.

### Myocardial infarction and heart failure

Injection of human pluripotent adipocyte derived mitochondria into cardiomyocytes and endothelial cells surrounding the infarct zone increased heme oxygenase-1 expression and mitochondrial biogenesis [82]. MSCs-based mitochondrial transplantation opens a new avenue for the use of mitochondrial transplantation in the treatment of myocardial infarction.

A key pathophysiological mechanism of heart failure is defective myocardial mitochondrial function. In end-stage myocardial failure, the activity of citrate synthase of complex I of the mitochondrial respiratory chain was reduced by 28%, therefore, mitochondrial transplantation holds extensive promise in the treatment of heart failure. Studies showed that autologous mitochondrial transplantation from rat skeletal muscle cells to cardiomyocytes improved cellular respiration and energy production in a short time [44]. Injection autologous calf muscle mitochondria into the right ventricular free wall in a porcine model of right ventricular hypertrophy/failure prolonged the physiological adaptation of the right ventricle under pressure load and maintained contractile ability by reducing cardiomyocytes apoptosis [83]. In doxorubicin induced heart failure, transfer of M2-like macrophages reduced cardiac fibrosis and cardiomyocyte apoptosis to improve cardiac function, which may be related to mitochondrial transfer, moreover, co-culture of M2 macrophages in vitro translocated mitochondria to cardiomyocytes and promoted repair of myocardial injury [84]. Transplantation of mitochondria with varying metabolic status, showing that metabolically matched mitochondria restored mitochondrial membrane potential and protected against doxorubicin-induced heart failure [85].

### Pulmonary hypertension

Pulmonary hypertension, a fatal progressive vascular disease, is caused by an increase in mean pulmonary artery pressure and right ventricular afterload due to pulmonary arteriolar occlusion, resulting in right ventricular hypertrophy and failure [86]. Two recent studies and other literature found that mitochondrial transplantation did not change the survival rate of animals [87, 88]. Mitochondrial transplantation in the experimental model of pulmonary hypertension provided beneficial effects in reducing pulmonary artery smooth muscle cell proliferation and pulmonary vasoconstriction, reducing pulmonary vascular remodeling, and improving right ventricular function [86–88]. Transplantation of mitochondria extracted from rat femoral artery smooth muscle cells into rat pulmonary artery smooth muscle cells by intravenous administration inhibits pulmonary vasoconstriction induced by acute hypoxia and attenuates pulmonary vascular remodeling induced by chronic hypoxia [87]. After mitochondria were isolated from immature rat soleus muscle and intravenously injected into pulmonary hypertensive rats, right ventricular mass and wall thickness returned to normal, and serum B-type natriuretic peptide levels and ventricular diameter were reduced, demonstrating that mitochondrial transplantation increased lung tissue ATP concentration, reversed pulmonary artery remodeling and improved right ventricular function [88].

### Ischemic stroke

Ischemic stroke, a life-threatening disease caused by a sudden decrease in cerebral blood flow followed by a decrease in oxygen and glucose, is associated with mitochondrial dysfunction [89]. The transfer of mitochondria from endothelial progenitor cells to ischemic brain endothelial cells showed increased mitochondrial biogenesis and mitochondrial DNA copy number, restored ATP level after ischemic stroke [89]. Many studies have also shown that human MSCs reduce inflammation and promote vascular growth and neurite outgrowth as well as functional recovery [90]. Interestingly, MSCs based mitochondrial transplantation is a novel treatment for ischemic stroke by promoting the bioenergetic profile of neuron and neurite regeneration, enhancing angiogenesis, reducing infarct volume, and improving functional recovery in cerebral ischemic rats [90].

In view of the fact that (i) the source of mitochondria used in most studies is autologous mitochondria and (ii) mitochondrial haplotypes may have a great impact on genome expression, which is related to mitochondrial-nuclear incompatibility and the spread of harmful mutations, these technical challenges and ethical issues greatly limit the clinical use of mitochondrial transplantation [4, 44]. According to the current literature, mitochondrial transplantation has only studied the problems related to ischemic heart injury in clinical treatment.

In the first clinical research on mitochondrial transplantation, autologous mitochondria from non-ischemic skeletal muscle were isolated and pericardial injection was given to pediatric patients with I/R injury supported by extracorporeal membrane oxygenation (ECMO), it showed that mitochondrial therapy did not cause inflammation or rejection but instead improved ventricular function, while the conclusion was statistically significant due to the limited sample size [91]. In addition, mitochondrial transplantation resulted in significantly shorter ventricular strain and adverse cardiovascular events and fewer adverse cardiovascular events. This suggests the promise of mitochondrial transplantation in the treatment of cardiogenic shock in pediatric patients with I/R injury [38]. In 2021, the McCully team used autologous mitochondrial transplantation again in the treatment of refractory cardiogenic shock in children [92]. Autologous mitochondrial transplantation in ECMO patients with cardiogenic shock favored successful isolation of ECMO with enhanced ventricular strain, further reflecting the great potential of mitochondrial transplantation in clinical therapy.

## Opportunities and challenges

### The advantages of mitochondrial transplantation

Intercellular mitochondrial transfer plays an important role under both physiological and pathological conditions. It occurs spontaneously under physiological conditions and regulates the development of the cardiovascular system by promoting the proliferation and differentiation of stem cells into cardiomyocyte through progenitor cells reprogramming [4, 7]. Intercellular mitochondrial transfer contributes to the release of dysfunctional mitochondria under pathological conditions. Intercellular mitochondrial transmission can also be sensed by other cells, for example, the release of dysfunctional mitochondria in I/R injury was sensed by MSCs to enhance mitochondrial biogenesis as a negative feedback mechanism [53]. Intercellular mitochondrial transfer also rescued cellular injury by taking up functional mitochondria, thereby improving mitochondrial biogenesis [65].

A great advantage of mitochondrial transplantation over traditional mitochondria targeted drug delivery therapies lies in: (1) Achieve better therapeutic effects on multiple targets (2) Etiological treatments which are equivalent to gene level (3) More benefits from a single injection (4) The switch of cell-based therapy to cell-free therapy will greatly improve the convenience and cost of mitochondrial transplantation. At the same time, controversy still exists on mitochondrial transplantation technology, and there is still a long way to go to achieve its widespread clinical application.

### Challenges and future prospective

In recent years, with the increasing popularity of mitochondrial transplantation, controversy over this treatment method has also emerged, mainly included in the following aspects.

Firstly, skeptics point out that McCully’s team did not rule out the possibility that mitochondria might be destroyed under injection conditions [93]. The concentration of calcium ions in the extracellular fluid is much higher than that in the intracellular fluid, whereas mitochondria are highly permeable to calcium ions, and the opening of the mPTP under high calcium conditions leads to mitochondrial inner membrane barrier disruption, mitochondrial permeability edema, inner membrane rupture, and even cell death. Although some earlier studies found mitochondria to be damaged in high calcium environments, this does not mean that they cannot survive at all. McCully team also discovered free mitochondria in the blood, however its activity may be normal or impaired [40, 94].

In addition, McCully’s group found that mitochondrial transplantation during reperfusion can improve cardiac function in as little as a few minutes [32], whereas mitochondria need to pass the endothelial barrier to integrate in the vicinity of cardiomyocytes, a process that takes several hours. And without a cytoplasmic environment, the conversion of glucose and fatty acids into pyruvate and fatty acyl coenzymes does not reveal how mitochondria produce ATP and complete cell contraction [93], this is also a very important aspect that needs to be clarified.

Furthermore, although McCully and colleagues found that mitochondrial transformation is feasible in cultured cells, whether mitochondria survive or not and are taken up by cardiomyocytes has not been clearly demonstrated, they entered cardiomyocytes but in low numbers [38], so the capacity to provide ATP might be limited. And studies showed that direct administration of exogenous ATP cannot significantly alleviate myocardial injury [37], in order to ensure the effect of mitochondrial transplantation, there must be sufficient mitochondria through the cell membrane to promote host cells to produce ATP. In fact, the immunogenicity of mitochondria is also closely related to transplantation efficiency. How to ensure that exogenous mitochondria are not rejected by individuals or phagocytosed by immune responses is also one of the very important issues.

It has become a consensus that mitochondria can be transferred between cells. Significant cellular endocytosis also appears from indirect or direct co incubation of isolated mitochondria with cells in in vitro experiments, but the mechanism of internalization after contact with tissue cells is not fully understood. What’s more, there is no definitive research evidence and conclusion as to whether there are differences in efficiency and efficacy when delivered in different ways.

Certainly, the timing and conditions of mitochondrial transplantation as a therapeutic approach can be further explored. The first step of mitochondrial transfer technically is the isolation of mitochondria, ensuring complete separation and good storage of mitochondria. Meanwhile, the core issue in mitochondrial transplantation is how to target and deliver mitochondria to specific tissues or organs. Therefore, future studies should focus on developing carriers for specific cellular delivery to improve the efficiency of mitochondrial internalization.

Finally, the effects of mitochondrial transplantation need to be further validated and evaluated in vivo. Delivery methods should be optimized according to the cell or tissue type and put into clinical practice.

## Summary

Mitochondria participate in a series of important cellular processes and are the metabolic center and signal platform of cells. Mitochondrial dysfunction is one of the major pathological features of CVD. A number of preclinical trials have demonstrated significant advantages of mitochondrial transplantation over traditional approaches to improve mitochondrial function in CVD treatment. Although the study of mitochondrial transplantation is in its infancy and many issues remain to be unanswered, there is no doubt that this new technology will have a promising future.

## Data Availability

Not applicable.

## Abbreviations

AMP: Adenosine monophosphate
AMPK: AMP-activated protein kinase
ATP: Adenosine triphosphate
MSCs: Mesenchymal stromal cells
CDC42: Cell division control protein 42 homolog
CVD: Cardiovascular disease
ECMO: Extracorporeal membrane oxygenation
EVs: Extracellular vesicles
hBM-MSCs: Human bone marrow mesenchymal stromal cells
hMADS: Human multipotent adipose-derived stem cells
iPSC-MSCs: Pluripotent stem cell-derived mesenchymal stem cells
I/R: Ischemia reperfusion
Miro1: Mitochondrial Rho GTPase 1
mPTP: Mitochondrial permeability transition pore
MSCs: Mesenchymal stem cells
mtDNA: Mitochondrial deoxyribonucleic acid
mTOR: Mammalian target of rapamycin
NAD: Nicotinamide adenine dinucleotide
ROS: Reactive oxygen species
TNTs: Tunnelling nanotubes
